# Profibrotic potential of Prominin-1^+ ^epithelial progenitor cells in pulmonary fibrosis

**DOI:** 10.1186/1465-9921-12-126

**Published:** 2011-09-26

**Authors:** Przemyslaw Blyszczuk, Davide Germano, Sokrates Stein, Holger Moch, Christian M Matter, Beatrice Beck-Schimmer, Thomas F Lüscher, Urs Eriksson, Gabriela Kania

**Affiliations:** 1 Cardiovascular Research and Zürich Center for Integrative Human Physiology; Institute of Physiology, University of Zürich, Winterthurerstr. 190, CH-8057 Zürich, Switzerland; 2 Department of Medicine, GZO - Zürich Regional Health Center, Spitalstr. 66, CH-8620 Wetzikon, Switzerland; 3 PreClinical Safety, Novartis Pharma AG, Klybeckstr. 141, CH-4057 Basel, Switzerland; 4 Departament of Pathology, University Hospital Zürich, Raemistr. 100 CH-8001 Zürich, Switzerland; 5 Departament of Cardiology, University of Zürich, Winterthurerstr. 190, CH-8057 Zürich, and University Hospital Zürich, Raemistr. 100, CH-8001 Zürich, Switzerland; 6 Lung Immunopathology, University of Zürich, Winterthurerstr. 190, CH-8057 Zürich, and University Hospital Zürich, Raemistr. 100, CH-8001 Zürich, Switzerland

**Keywords:** bone marrow, idiopathic pulmonary fibrosis, lung, myofibroblasts, progenitor, prominin-1/CD133

## Abstract

**Background:**

In idiopathic pulmonary fibrosis loss of alveolar epithelium induces inflammation of the pulmonary tissue followed by accumulation of pathogenic myofibroblasts leading eventually to respiratory failures. In animal models inflammatory and resident cells have been demonstrated to contribute to pulmonary fibrosis. Regenerative potential of pulmonary and extra-pulmonary stem and progenitor cells raised the hope for successful treatment option against pulmonary fibrosis. Herein, we addressed the contribution of lung microenvironment and prominin-1^+ ^bone marrow-derived epithelial progenitor cells in the mouse model of bleomycin-induced experimental pulmonary fibrosis.

**Methods:**

Prominin-1^+ ^bone marrow-derived epithelial progenitors were expanded from adult mouse lungs and differentiated *in vitro *by cytokines and growth factors. Pulmonary fibrosis was induced in C57Bl/6 mice by intratracheal instillation of bleomycin. Prominin-1^+ ^progenitors were administered intratracheally at different time points after bleomycin challenge. Green fluorescence protein-expressing cells were used for cell tracking. Cell phenotypes were characterized by immunohistochemistry, flow cytometry and quantitative reverse transcription-polymerase chain reaction.

**Results:**

Prominin-1^+ ^cells expanded from healthy lung represent common progenitors of alveolar type II epithelial cells, myofibroblasts, and macrophages. Administration of prominin-1^+ ^cells 2 hours after bleomycin instillation protects from pulmonary fibrosis, and some of progenitors differentiate into alveolar type II epithelial cells. In contrast, prominin-1^+ ^cells administered at day 7 or 14 lose their protective effects and differentiate into myofibroblasts and macrophages. Bleomycin challenge enhances accumulation of bone marrow-derived prominin-1^+ ^cells within inflamed lung. In contrast to prominin-1^+ ^cells from healthy lung, prominin-1^+ ^precursors isolated from inflamed organ lack regenerative properties but acquire myofibroblast and macrophage phenotypes.

**Conclusion:**

The microenvironment of inflamed lung impairs the regenerative capacity of bone marrow-derived prominin-1^+ ^progenitors and promotes their differentiation into pathogenic phenotypes.

## Introduction

Any tissue injury triggers inflammation, a complex pathophysiological process, supposed to attenuate injury, and to induce reparative processes. However, exaggerated inflammatory responses may exacerbate tissue damage, and result in excessive scarring further compromising organ function. Idiopathic pulmonary fibrosis (IPF) is a lung disease of unknown origin characterized by loss of lung epithelial cells and pathological parenchymal tissue remodelling, which results in accumulation of myofibroblasts, distortion of lung architecture, and eventually respiratory failure [1]. Prognosis of IPF patients is poor and effective therapeutic options are lacking [2].

Bleomycin-induced experimental pulmonary fibrosis is the best-characterized animal model in use today [3]. Intratracheal instillation of bleomycin results in oxidative damage to the alveolar epithelium and the recruitment of inflammatory cells. After resolution of the acute inflammation, a chronic fibrotic process develops, which is characterized by replacement of extracellular matrix by fibrillar collagen and collagen-producing fibroblasts and myofibroblasts. However, the molecular and cellular mechanisms remain unclear.

Formation of type I collagen-producing, alpha smooth muscle actin (αSMA)-positive myofibroblasts is a hallmark of pulmonary fibrosis. Despite decades of extensive research, the origin of pulmonary myofibroblasts remains elusive. Transformation of parenchymal epithelial cells into myofibroblasts through epithelial-to-mesenchymal transition is currently considered as a major process in the development of pulmonary fibrosis [4,5]. However, other studies point to stromal fibroblasts and bone marrow-derived cells as important sources of pulmonary myofibroblasts [5-7]. Of note, in bleomycin-induced experimental pulmonary fibrosis pathological fibroblasts originate from different cellular sources [8].

Stem and progenitor cells represent a potentially attractive treatment option against pulmonary fibrosis. Several studies reported that lungs indeed contain pools of endogenous pulmonary stem and progenitor cells [9-11]. Furthermore, bone marrow-derived stem and progenitor cells isolated from the lung [11,12] or from other tissues [13-15] have the capacity to differentiate into pulmonary epithelial cells. In addition, these cells exhibit anti-inflammatory properties when administrated early at the onset of the disease [12,16]. Nevertheless, bone marrow-derived cells contribute only marginally to lung regeneration [17,18] and we do not know yet, how the specific microenvironment of the diseased lungs alters fate and function of endogenous or therapeutically administered stem and progenitor cells.

Prominin-1 (CD133) is a membrane-associated glycoprotein present on hematopoietic stem and progenitor cells [19,20]. Recently, we have described bone marrow-derived lung resident prominin-1^+ ^epithelial progenitors with immunosuppressive capacity and their ability to differentiate into alveolar type II epithelial cells [12]. Herein, using a mouse model of bleomycin-induced experimental lung injury we analysed the properties of the prominin-1^+ ^epithelial progenitor cells in the lungs undergoing fibrotic remodelling.

## Material and Methods

### Mice

C57Bl/6 mice and C57Bl/6-enhanced green fluorescent protein (EGFP) transgenic mice (EGFP under control of β-actin promoter) were purchased from Jackson Laboratory. All animal experiments were conducted in accordance with institutional guidelines and Swiss federal law and were approved by the local authorities.

### Generation of bone marrow chimera

5-7-week-old C57Bl/6 mice were lethally irradiated with two doses of 6.5 Gy using a Gammatron (Co-60) system and reconstituted with 2x10^7 ^donor bone marrow cells from C57Bl/6-EGFP mice.

### Induction of bleomycin-induced lung fibrosis and treatment protocols

7-9-week-old C57Bl/6 or 11-13-week-old C57Bl/6-EGFP chimera mice were anesthetized and intratracheally injected with 0.05 U/mouse of bleomycin (Blenoxane, Axxora-Alexis) as described [12]. In the respective experiments, the animals received intratracheally 2 × 10^5 ^prominin-1^+ ^cells 2h, 24h, 3d, 7d or 14d after bleomycin instillation.

### Cell culture

Cells were isolated from mouse lungs as described previously [12]. Prominin-1^+ ^cells were expanded in the culture expansion medium (CEM; Additional file 1). In the respective experiments, magnetic cell sorting using anti-prominin-1-PE antibody (eBioscience) and anti-PE magnetic beads (Miltenyi) was used to enrich population of prominin-1-expressing cells. To generate single cell derived clones, 1-5 prominin-1^+^/EGFP^+ ^cells were co-plated with prominin-1^+^/EGFP^- ^feeder cells derived from the healthy lung, and cultured for 2-3 weeks. Type II lung alveolar epithelial differentiation was induced in the presence of the modified Small Airway Growth Medium (SAGM; Cambrex) as described previously [12]; macrophage differentiation with 10 ng/mL macrophage-colony stimulating factor (M-CSF, PeproTech); and fibroblast differentiation with 10 ng/mL TGF-β (PeproTech) as described before [21].

### Reverse transcription and quantitative polymerase chain reaction

RNA isolation and cDNA synthesis were performed as described [22]. cDNA was amplified using the Power SYBR Green PCR Master Mix (Applied Biosystems) and oligonucleotides complementary to transcripts of the analyzed genes (Additional file 1).

### Histology, immunocytochemistry and phagocytosis assay

Formalin-fixed, paraffin-embedded lung sections were stained with hematoxylin and eosin for histological analysis and with Masson's trichrome staining for detection of collagen fibers. Immunofluorescence analysis was performed on frozen tissue sections and cells cultured on gelatin-coated cover slips as described previously [12]. For prominin-1 detection, frozen sections and cultured cells were stained with the appropriate primary and followed with secondary antibody (Additional file 1) prior to fixation with 4% paraformaldehyde. Phagocytosis activity assay was performed using the Alexa Fluor 488- or Texas Red-conjugated *E. coli *BioParticles (Invitrogen) according to manufacture's recommendations.

### Western-blot

Prominin-1^+ ^cells were challenged with TGF-β (PeproTech) for 1, 6 and 24 hours. Control cells were cultured in the absence of TGF-β Cell lysates were blotted and incubated with appropriate antibodies (Additional file 1).

### Flow cytometry

Cells were filtered through 70-μm nylon mesh filter, stained for 30 minutes on ice with the appropriate antibodies (Additional file 1), and analyzed on a CyAN ADP (Dako-Cytomation) using FlowJo 8.7.3 software (TreeStar).

### Statistics

Normally distributed data were compared using Student *t *test or 1-way ANOVA followed by Bonferroni's post-test. Statistical analysis was conducted using Prism 4 software (GraphPad Software). Differences were considered as statistically significant for p < 0.05.

## Results

### Prominin-1^+ ^expression characterizes epithelial progenitors with multilineage differentiation capacity

**Figure 1:**
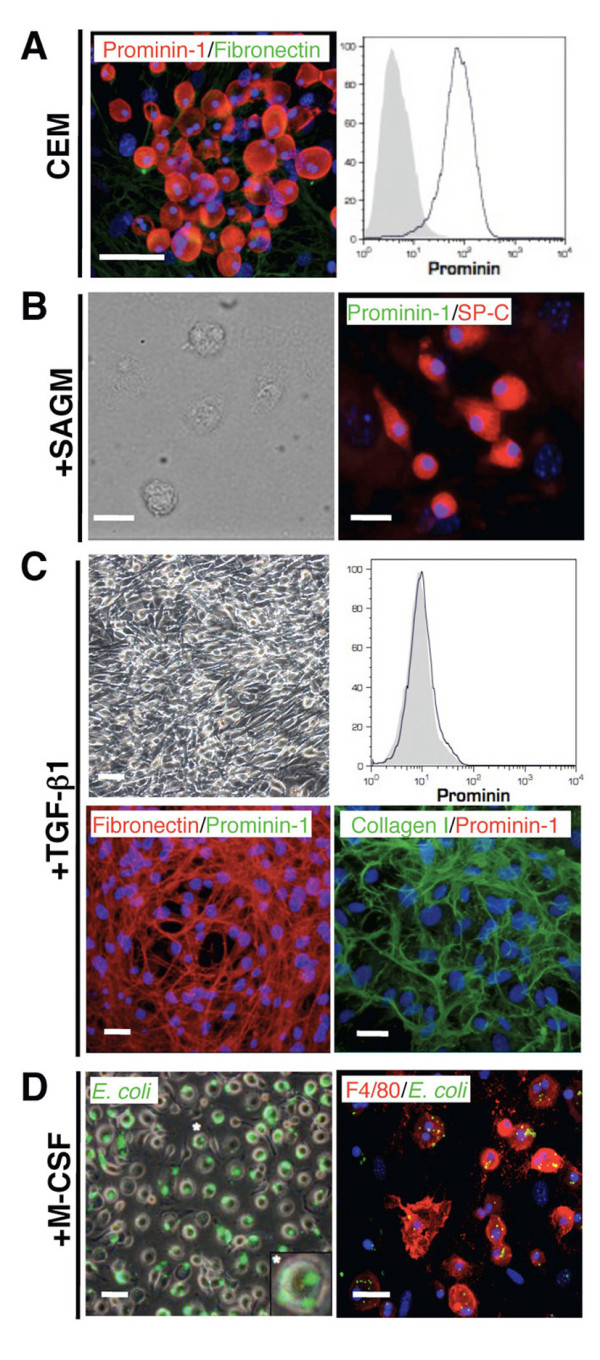
**Lung-derived prominin-1^+ ^cells turn into alveolar type II epithelial cells, fibroblasts or macrophages after exposure to different cytokines and growth factors**. Expansion of cells from the healthy lung explants in the culture expansion medium (CEM) resulted in round, semi-adherent prominin-1-positive cells and fibronectin-positive feeder layer (A, left). Harvested cells contained mostly prominin-1-positive cells (A, right). Further, prominin-1-positive cells were isolated using magnetic cell sorting and cultured in the presence of different cytokines and growth factors. Prominin-1^+ ^cells cultured in the Small Airway Growth Medium (SAGM) for 14 days became surfactant protein-C (SP-C)-positive and prominin-1-negative (B). Prominin-1^+ ^cells cultured in the presence of TGF-β for 14 days lost prominin-1 expression, but instead produced fibronectin and collagen I (C). Exposure of prominin-1^+ ^cells to M-CSF for 7 days resulted in formation of *E.coli *phagocytising F4/80-positive cells (D). DAPI visualized cell nuclei. Bars = 20 μm.

We have previously demonstrated that bone marrow-derived prominin-1^+ ^cells expanded from healthy lung explants represent progenitors of alveolar type II epithelial cells [12]. In order to investigate if these prominin-1^+ ^progenitors differentiate into other cell types typically observed in IPF, such as myofibroblasts and macrophages, we expanded prominin-1^+ ^cells from lung tissue in the culture expansion medium (CEM) [12]. Expanded prominin-1^+ ^cells were negative for fibronectin (Figure 1A) and other lineage-specific markers (data not shown), but expressed CD45, c-kit, Sca-1 and Cxcr4 [12]. Next, we sorted prominin-1-expressing cells and induced differentiation towards type II pneumocytes, fibroblasts and macrophages. Two weeks of culture in the modified Small Airway Growth Medium (SAGM) resulted in formation of pulmonary type II cells positive for surfactant protein-C (SP-C; Figure 1B) as described [12]. In contrast, prominin-1^+ ^cells cultured in the presence of TGF-β differentiated into fibronectin- and collagen I-producing fibroblast (Figure 1C). Furthermore, addition of M-CSF to the culture medium resulted in the formation of F4/80^+ ^macrophages (Figure 1D).

**Figure 2:**
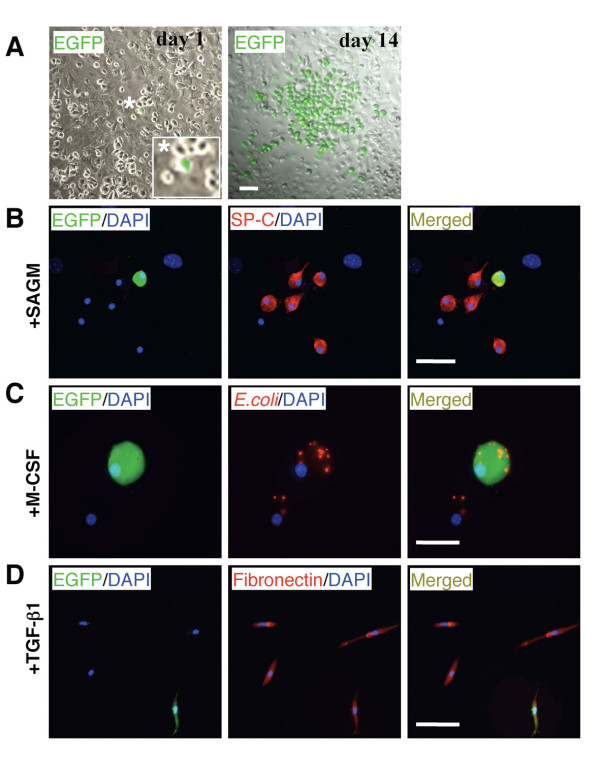
**Lung-derived prominin-1^+ ^cells represent a common progenitor of alveolar type II epithelial cells, fibroblasts and macrophages**. Prominin-1-positive cells were isolated from lungs of C57Bl/6-EGFP mouse. One EGFP-expressing prominin-1^+ ^cell co-cultured with cells from healthy lungs for 14 days in the CEM proliferated and generated EGFP-positive single cell-derived clone (A). Co-culture containing the EGFP-positive single cell-derived clone were split and cultured under conditions stimulating different lineage differentiation. After 7-14 days, EGFP-expressing cells were positive for SP-C in the SAGM (B), phagocyted *E.coli *after treatment with M-CSF (C), and were positive for fibronectin in the presence to TGF-β (D). DAPI visualized cell nuclei. Bars = 20 μm.

Next, we addressed whether prominin-1^+ ^cells represent a common progenitor for alveolar type II epithelial cells, fibroblasts and macrophages. We expanded prominin-1^+ ^cells from lung explants of C57Bl/6-EGFP mouse and plated 1-5 sorted EGFP^+^/prominin-1^+ ^cells on non-transgenic lung-derived feeder layer. After 14 days, we observed single cell-derived EGFP^+ ^colonies (Figure 2A). Thereafter, co-cultures containing the single EGFP^+ ^clone were divided into three different conditions stimulating the lineage differentiation described above. EGFP^+ ^cells differentiated into SP-C-positive type II pneumocytes in the SAGM medium (Figure 2B), phagocyting macrophages after exposure to M-CSF (Figure 2C), and fibronectin-positive fibroblasts in response to TGF-β (Figure 2D). We differentiated successfully 10 single cell-derived clones. Taken together, these data demonstrate that all three phenotypes can originate from a single prominin-1^+ ^progenitor. Thus, prominin-1 expression on bone marrow-derived cells in the adult mouse lung is specific for multilineage progenitors.

### Bleomycin-induced pro-fibrotic pulmonary microenvironment affects the fate of prominin-1^+ ^cells

**Figure 3:**
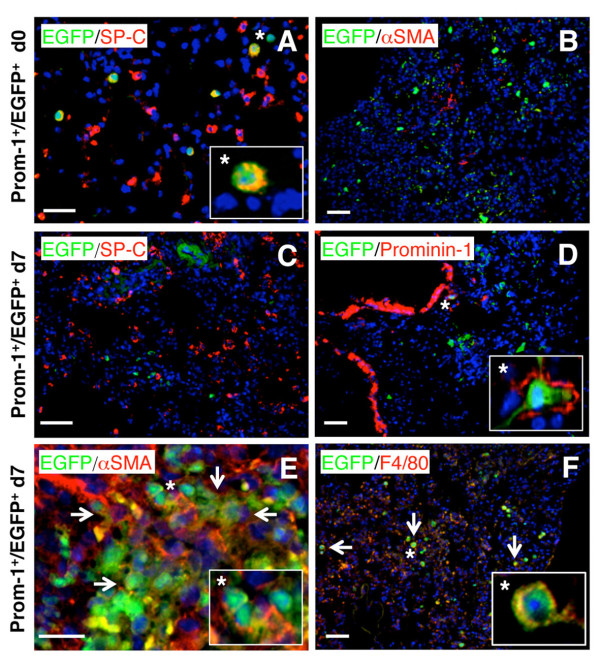
**Differentiation of prominin-1^+ ^progenitors is stage-specific in bleomycin-challenged mice**. Expanded EGFP^+ ^prominin-1-positive cells were isolated using magnetic cell sorting and transplanted into C57Bl/6 mice by intratracheal injection 2 hours or 7 days after bleomycin challenge. Some of engrafted EGFP^+ ^cells injected 2 hours after bleomycin treatment were SP-C-positive (A), but nearly all were negative for αSMA 7 days after transplantation (B). Instead, EGFP^+^/prominin-1^+ ^cells transplanted 7 days after bleomycin treatment (into the lung with ongoing inflammation) and analyzed at day 21 were negative for SP-C (C), rarely positive prominin-1 (D), but some expressed αSMA (E) and F4/80 (F). DAPI visualized cell nuclei. Bars = 20 μm.

Given the high *in vitro *plasticity of prominin-1^+ ^progenitors, we tested how changes in the pulmonary microenvironment, which parallels fibrosis progression affect their differentiation capacity *in vivo *within the affected organ. Thus, we expanded cells from healthy lung of C57Bl/6-EGFP^+ ^mouse and instilled sorted prominin-1^+ ^progenitors intratracheally 2h and 7d after bleomycin challenge to C57Bl/6 recipients. Lungs of recipient mice were analyzed 1 or 2 weeks after engraftment of the EGFP^+ ^cells. Injection of prominin-1^+^/EGFP^+ ^cells 2h after bleomycin instillation protected from pulmonary inflammation and fibrosis at day 7 (not shown; [12]). At this time point, we found some EGFP^+ ^cells positive for SP-C (Figure 3A), but nearly all were negative for the myofibroblast-specific marker αSMA (Figure 3B). In contrast, prominin-1^+^/EGFP^+ ^cells administrated to lungs with active inflammation (7d after bleomycin instillation) failed to express SP-C (Figure 3C), and mostly lost prominin-1 expression (Figure 3D), but instead were positive for αSMA (Figure 3E) and F4/80 (Figure 3F) within the lung tissue analysed at day 21. Taken together, the specific microenvironment of the inflamed or fibrotic lung determines the fate of transplanted multilineage prominin-1^+ ^progenitors.

**Figure 4:**
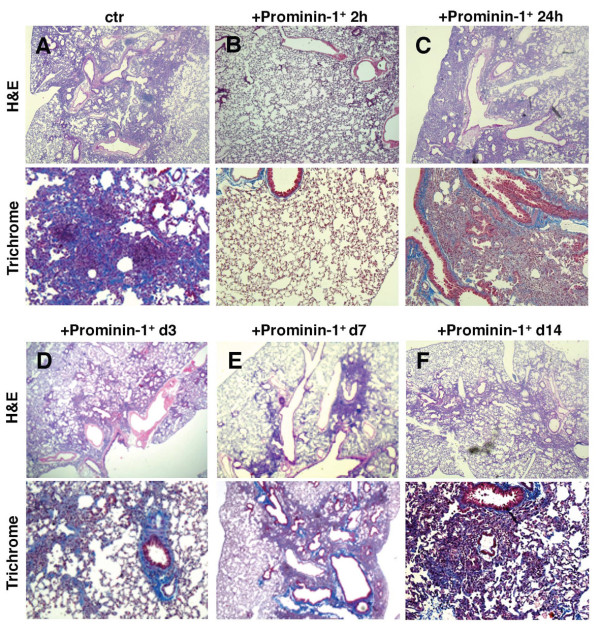
**Attenuation of bleomycin-induced pulmonary fibrosis by prominin-1^+ ^cells is stage-specific**. Expanded prominin-1-positive cells were isolated using magnetic cell sorting and transplanted into recipient mice by intratracheal injection at different time points after bleomycin instillation. Lung sections were analyzed 21 days after bleomycin instillation by hematoxylin and eosin (H&E) for excessive non-parenchymal infiltrates and Masson's trichrome staining for collagen I deposition (blue). Control mice injected with bleomycin developed severe pulmonary fibrosis (A). Administration of prominin-1^+ ^cells 2 hours after bleomycin treatment protected the mice from fibrosis (B). Mice receiving prominin-1^+ ^cells 24 hours (C), 3 days (D), 7 days (E) or 14 days (F) after bleomycin challenge failed to protect from pulmonary fibrosis. Magnifications: x100. Microphotographs from one independent experiment are shown for each time point. For quantification see in Additional file 1, Figure S1.

Next, we analyzed how prominin-1^+ ^cells affect bleomycin-induced pulmonary fibrogenesis at different time points of the disease progression. We administrated prominin-1^+ ^progenitors 2h, 24h, 3d, 7d and 14d following bleomycin instillation and analyzed the extent of pulmonary fibrosis at day 21. Histological analysis of lung sections revealed that prominin-1^+ ^progenitors are only protective if they were injected within 2 h after bleomycin instillation (Figure 4A, B; Additional file 1, Figure S1). In contrast, prominin-1^+ ^progenitors delivered after 24h, 3d, 7d or 14d failed to attenuate bleomycin-induced fibrosis (Figure 4C-F; Additional file 1, Figure S1). These findings indicate that the changing pulmonary microenvironment at different stages of pulmonary fibrosis affects the anti-inflammatory properties of prominin-1^+ ^cells.

### Bleomycin promotes the accumulation of prominin-1^+ ^progenitors in the injured lung

**Figure 5:**
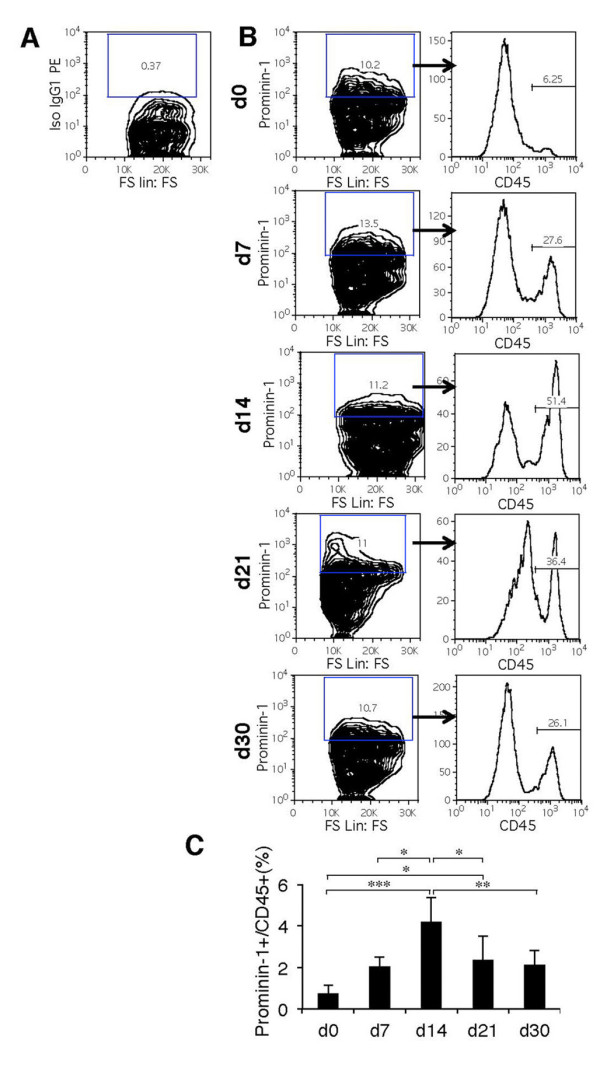
**Bone marrow-derived prominin-1^+ ^cells accumulate in the lung of bleomycin-instilled mice**. Flow cytometry analysis of CD45 expression gated on prominin-1^+ ^cells in the lung before (d0) and 7, 14, 21 and 30 days after bleomycin instillation. Density plots and histograms demonstrate one representative out of five independent experiments (A, B). Quantification of flow cytometry analysis of prominin-1^+^/CD45^+ ^cells out of all analyzed cells in the lung tissue following bleomycin treatment (C). FS - forward scatter, Iso- isotype control. Bars represent mean ± SD from 5 individual lung tissues.

Inflammation mobilizes and activates local or exogenous stem and progenitor cells. We therefore investigated whether bleomycin instillation promotes the accumulation of bone marrow-derived prominin-1^+ ^progenitors in the lung. So far, we have identified two distinct populations of prominin-1 expressing cells in the healthy lung. Bone marrow-derived prominin-1^+lo ^progenitors were identified as CD45-expressing cells with non-polarized membrane distribution of the prominin-1 antigen and represent about 7% of total prominin-1^+ ^cells in healthy lung tissue [12]. After bleomycin application, we observed increasing proportions of CD45 expression within the whole prominin-1^+ ^cell population (Figure 5A). 14 days after bleomycin instillation prominin-1^+^/CD45^+ ^cell subset increased about three fold compared to unaffected lungs (Figure 5B). Furthermore, immunofluorescence analysis revealed increased amount of cells with non-polarized prominin-1 localization on the cellular membrane during the inflammatory phase (d7) and accumulation of αSMA^+ ^myofibroblasts during disease progression (Additional file 1, Figure S2). Increased bone marrow-derived prominin-1^+ ^progenitors within the inflamed and fibrotic lungs suggest an active contribution of this cell subpopulation to the development of bleomycin-induced experimental pulmonary fibrosis.

### Bone marrow-derived cells enhance bleomycin-induced experimental pulmonary fibrosis

**Figure 6:**
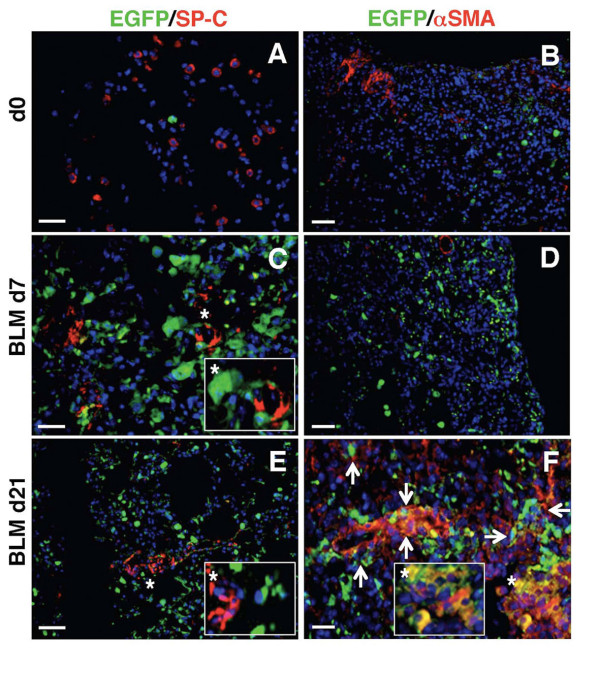
**Bone marrow-derived myofibroblasts contribute to bleomycin-induced pulmonary fibrosis**. C57Bl/6 mice were lethally irradiated and reconstituted with bone marrow of syngeneic C57Bl/6-EGFP animals. 6 weeks after bone marrow reconstitution, some of the chimeric mice received bleomycin to induce pulmonary fibrosis. In the lung of unchallenged chimeric mice, EGFP-positive cells were negative for SP-C (A) and αSMA (B). In the chimeric mice 7 days after bleomycin instillation, EGFP-positive cells accumulated in the inflamed lung tissue but were negative for SP-C (C) and αSMA (D). 21 days after treatment with bleomycin, some EGFP^+ ^cells were positive for αSMA (F), but not for SP-C (E). DAPI visualized cell nuclei. Bars = 20 μm.

Accumulation of αSMA^+ ^and collagen I^+ ^myofibroblasts is a hallmark of pathological remodelling in pulmonary fibrosis. Next, we analyzed the contribution of bone marrow-derived cells to regenerative SP-C^+ ^alveolar type II epithelial cells and pathological αSMA^+ ^myofibroblasts in our model. We lethally irradiated C57Bl/6 mice and reconstituted them with C57Bl/6-EGFP syngeneic bone marrow. Six weeks after bone marrow reconstitution, we found no EGFP^+ ^cells co-expressing αSMA or SP-C in the lung of the chimeric mice (Figure 6A-B; d0). After bleomycin instillation, acute lung inflammation developed (d7), and at this stage we observed no evident αSMA and SP-C expression in EGFP^+ ^cells (Figure 6C, D). Instead, accumulated EGFP^+ ^inflammatory cells expressed prominin-1 (around 30-40%; Additional file 1, Figure S3A). Analysis of fibrotic lungs (d21) demonstrated that around some EGFP^+ ^cells became αSMA^+ ^myofibroblasts, but not SP-C^+ ^type II epithelial or β-tubulin IV-expressing cells (Figure 6E-F; Additional file 1, Figure S3B) in the chimeric mice. αSMA^+ ^fibroblasts contain around 25-30% of EGFP-expressing cells (calculation not shown). Of note, pulmonary fibrosis in chimeric mice was comparable to C57Bl/6 mice (Additional file 1, Figure S4). These findings indicate that in mouse model of bleomycin-induced experimental pulmonary fibrosis, bone marrow represents one of the cellular sources for progenitor cells differentiating into myofibroblasts, but not type II pneumocytes.

### Prominin-1^+ ^progenitors isolated from diseased lungs display impaired *in vitro *regenerative potential

**Figure 7:**
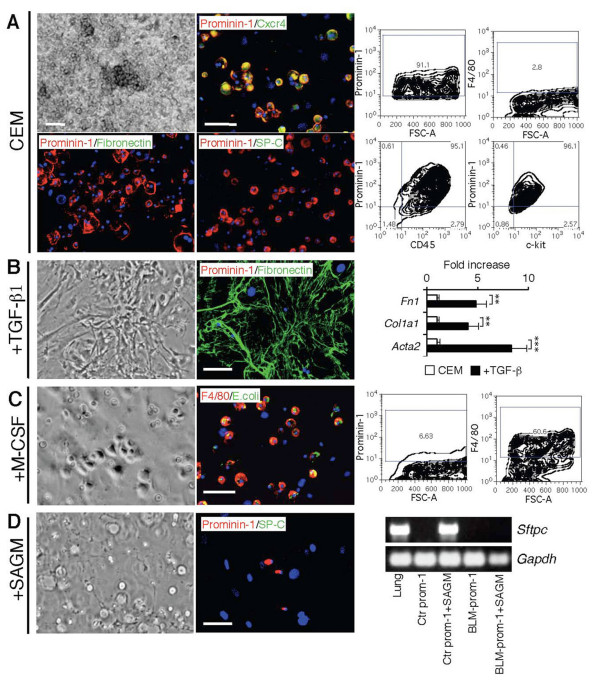
**Prominin-1^+ ^cells expanded from lungs of bleomycin-treated mice fail to acquire phenotype of type II alveolar epithelial cells**. Seven days after treatment with bleomycin, lungs with ongoing inflammation of C57Bl/6 mice were dissected and prominin-1-positive cells were isolated using magnetic cell sorting and cultured in the culture expansion medium (CEM) for 2-3 weeks. Expanded cells showed a round, semi-adherent phenotype, were positive for prominin-1, CD45, CXCR4, c-kit, and mostly negative for F4/80, SP-C and fibronectin (A). Further, sorted prominin-1^+ ^cells were stimulated for different lineage differentiation. In the presence of TGF-β prominin-1^+ ^progenitors formed fibronectin-positive cells and up-regulated myofibroblast-specific mRNA levels: *Fn1, Col1a1 *and *Acta2 *(B). Bars represent mean ± SD from at least 5 individual cell cultures. Addition of M-CSF to cultures resulted in formation of *E.coli *phagocytising F4/80-positive cells (C). Prominin-1^+ ^cells derived from inflamed lungs cultured in the SAGM for 14 days failed to produce SP-C at protein and mRNA levels (D). Abbreviations: ctr prom-1- prominin-1^+ ^cells isolated from healthy lung, BLM-prom-1- prominin-1^+ ^cells isolated from the inflamed lung 7 days after bleomycin instillation. DAPI visualized cell nuclei. Bars = 20 μm. (**) - p < 0.01, (***) - p < 0.001.

Next, we addressed the differentiation potential of prominin-1^+ ^progenitors during acute pulmonary inflammation. We injected intratracheally bleomycin and, after 7 days, sorted prominin-1^+ ^cells from the inflamed lung and expanded them in the CEM medium for 2-3 weeks. This resulted in the expansion of small, round, highly proliferating cells expressing prominin-1, CD45, c-kit and CXCR4 antigens, but not type II epithelial cell-, macrophage- or myofibroblast-specific markers (Figure 7A). Of note, all prominin-1^+ ^cells following the *in vitro *culture co-expressed CD45 (Figure 7A), but not β-tubulin IV- a marker of bronchial epithelial cells (not shown). Thus, prominin-1^+ ^cells expanded from healthy and inflamed lungs show the same phenotypic characteristic [12]. Then, we addressed whether prominin-1^+ ^cells expanded from inflamed lungs differentiate into myofibroblast, macrophage, and type II pneumocyte cell lineages *in vitro*. Cells were again sorted for prominin-1 prior to differentiation induction. In the presence of TGF-β prominin-1^+ ^cells significantly up-regulated *Fn1, Col1a1 *and *Acta2 *mRNA expression, lost prominin-1 expression and acquired fibronectin-positive myofibroblast phenotype (Figure 7B). Furthermore, TGF-β stimulation activated Smad pathway on prominin-1^+ ^progenitors as demonstrated by phosphorylation of Smad2 (P-Smad2; Additional file 1, Figure S5). In the presence of M-CSF, on the other hand, prominin-1^+ ^cells lost prominin-1 expression and became F4/80^+ ^macrophages (Figure 7C). However, prominin-1^+ ^cells expanded from inflamed lungs failed to up-regulate *Sftpc *expression and did not differentiate towards SP-C-positive type II alveolar epithelial cells upon culture in the SAGM medium (Figure 7D). This is in strong contrast to prominin-1^+ ^progenitors derived from the healthy lungs (Figure 7D). Taken together, our data indicate that inflammatory processes in the lung impair regenerative capacity of bone marrow-derived prominin-1-expressing progenitors.

## Discussion

We recently identified a population of bone marrow-derived lung resident prominin-1^+ ^epithelial progenitor cells with the capacity to differentiate into alveolar type II epithelial cells *in vitro *and *in vivo *[12]. Here, we report that these cells represent a common progenitor for type II epithelial cells, macrophages and myofibroblasts. Furthermore, we show that lineage commitment of prominin-1^+ ^progenitors critically depends on epigenetic stimuli, such as cytokines or microenvironment in the lung.

Several studies reported the ability of bone marrow-derived cells to become lung epithelial cells in mouse [11-14] and in humans [23,24]. This notion nourished the hope for rapid development of regenerative cell-based therapies using easily accessible hematopoietic stem and progenitor cells. However, recent observations from transgenic animal models clearly demonstrated that naturally occurring regeneration from any cells of hematopoietic origin is minimal after lung injury [17,18]. Our study proposes a potential mechanism explaining this discrepancy. We suggest that pathophysiological processes in affected lungs promote commitment of progenitors into non-regenerative cell phenotypes, such as pathological macrophages or myofibroblasts. Lung during inflammation and fibrosis is characterized by distinct and stage-specific expression pattern of chemokines, cytokines, growth factors, and extracellular matrix structure, creating a specific pulmonary signalling milieu [1]. As previously reported, lungs of bleomycin-instilled mice show elevated levels of chemokines and pro-inflammatory cytokines one week after the bleomycin challenge, and prominent production of pro-fibrotic mediators, including TGF-β pulmonary fibrosis [12]. Our results demonstrate that individual cytokines *in vitro *and the stage-specific signalling in the lung determine the fate of multilineage progenitor cells. Our observations are in line with studies on irradiation-induced lung inflammation demonstrating that mesenchymal stem cells injected at early phase of lung injury differentiate into epithelial and endothelial cells, while those injected at a late stage acquired αSMA^+ ^myofibroblast phenotype [25]. Thus, we hypothesize that in mouse model of bleomycin-induced pulmonary fibrosis, progenitor cells become activated upon injury, however, the signalling in the affected lung promotes formation of non-regenerative cell phenotypes.

Furthermore, our results showed that administration of prominin-1^+ ^progenitors only 2 hours after bleomycin instillation prevents pulmonary fibrosis development. Instead, transplantation of prominin-1^+ ^progenitors during ongoing inflammation or fibrogenesis fails to attenuate disease progression. Of note, anti-inflammatory effects of mesenchymal stem cells were only observed when delivered immediately after bleomycin instillation [16,26,27]. We therefore suggest that lineage commitment induced by inflammatory and fibrotic environment can explain these observations. Accordingly, it is conceivable that differentiating cells produce less anti-inflammatory factors, such as nitric oxide for example, and lose their anti-inflammatory properties. However, we cannot exclude that efficient attenuation of ongoing inflammation or fibrosis requires simply higher number of transplanted cells for an adequate response.

In this study we demonstrated that bone marrow-derived cells, and in particular, prominin-1^+ ^progenitors represent one of the cellular sources for myofibroblasts in bleomycin-induced experimental pulmonary fibrosis. Our data are in line with a previous report showing the formation of bone marrow-derived fibroblasts in lungs of chimeric mice in response to bleomycin challenge [7]. Furthermore, bone marrow-derived fibroblasts and myofibroblasts have been found in other models of pulmonary disorders including irradiation-induced lung fibrosis [6], asthma [28], bronchopulmonary dysplasia [29], and even after paracetamol treatment [30]. So far, collagen I-producing CD45^+ ^circulating fibrocytes have been identified as an important cellular source for myofibroblasts of hematopoietic origin [31]. Prominin-1^+ ^cells share features of fibrocytes, such as expression of CD45, CXCR4, but are negative for collagen I and CD34, and therefore clearly represent a distinct cell population

Our data further point to central role of TGF-β pathway in conversion of prominin-1^+ ^cells into pathological myofibroblasts. This is not surprising, because TGF-β has been implicated in different fibrogenic processes in the lung. For example, organ-specific over-expression of TGF-β in the lung of adult mice is sufficient to induce pulmonary fibrosis [32]. On the cellular level, TGF-β signalling not only promotes myofibroblast lineage commitment, but also induces epithelial-to-mesenchymal transition of alveolar epithelial cells [33]. On the molecular level, TGF-β stimulates the synthesis and deposition of collagen I [2]. In our model, TGF-β signalling mediated the phosphorylation of Smad2 proteins, pointing to the involvement of canonical Smad-dependent signalling pathways [34,35] in the transition of progenitors into myofibroblasts.

Multipotent nature and uncontrollable *in vivo *lineage commitment of stem and progenitor cells raised serious questions about the safety of stem cell-based therapies against IPF. Furthermore, our findings highlight the need for careful evaluation of cells isolated from injured organs for cell-based therapies. It remains to be determined whether these mechanisms are specific for bone marrow-derived cells or affect also the function of "true" pulmonary epithelial stem and progenitor cells. Recently, it has been reported that transplantation of alveolar type II cells improves outcomes in bleomycin-induced fibrosis irrespective of the disease stage [36]. This finding opts for use of committed or differentiated cells in cell-based regenerative approaches. However, an attractive alternative is targeting stem and progenitor cells naturally residing in the affected lungs, in order to inhibit their contribution to pathological processes and even to re-activate their regenerative potential. Thus, hematopoietic stem and progenitor cells represent a powerful tool in regenerative medicine. However, in-depth understanding of stem cell biology and the nature of hematopoietic cells are required for successful cell-based therapy against IPF.

## Conclusions

Herein, we provide evidence that the pro-fibrotic microenvironment suppresses the regenerative capacity of prominin-1^+ ^progenitor cells, and instead promotes their differentiation into pathological myofibroblasts and macrophages. Furthermore, we show that prominin-1^+ ^progenitor cells derived from healthy or inflamed lung tissues differ in their regenerative capability. Therefore, we concluded that the microenvironment of injured lung tissue dictates the fate and function of bone-marrow-derived cell progenitors, which may either support pathological remodelling or actively contribute to regeneration in the lungs. Thus, our findings highlight the need for careful evaluation of cells isolated from injured organs for cell-based therapies.

## Competing interests

The authors declare that they have no competing interests.

## Authors' contributions

Conception and design: PB, DG, UE, GK. Analysis and interpretation: PB, DG, SS, CMM, HM, UE, GK. Drafting the manuscript for important intellectual content: PB, DG, BBS, TFL, UE, GK.

All co-authors have read and approved the final manuscript.

## Authors' information

Urs Eriksson and Gabriela Kania shared last authorship on this manuscript.

## Supplementary Material

Additional file 1 **Additional methods and figure legends**. The file contains the additional methodological information and the figure legends to the additional figures S1-S5. Click here for file
