# Supplementary material for: Profibrotic potential of Prominin-1+ epithelial progenitor cells in pulmonary fibrosis
Source: Respir Res. 2011 Sep 26;12(1):126. doi: 10.1186/1465-9921-12-126 (PMC3191512; doi:10.1186/1465-9921-12-126)
Supplement: Additional file 1 — Additional methods and figure legends. The file contains the additional methodological information and the figure legends to the additional figures S1-S5. [file 1465-9921-12-126-S1.PDF]

Additional file 1

Additional methods

Cell cultures

Cells were isolated from mouse lungs as described previously [1]. In the respective experiments, prominin-1^+ cells were isolated either form the healthy or inflamed lungs (7 days after BLM instillation) by magnetic cell sorting using anti-prominin-PE antibody (eBioscience) and anti-PE magnetic beads (Miltenyi Biotec). For expansion of prominin-1^+ progenitors, isolated cells were resuspended in the Culture Expansion Medium (CEM) containing Iscove's Modified Dulbecco's Medium, 2% foetal calf serum, 100 mM, b-mercaptoethanol, 100 U penicillin, 100 mg/mL streptomycin, 2mM L-glutamine, 25 mM, N-2-hydroxyethylpiperazine-N9-ethane sulfonic acid (all Invitrogen), and plated at $5x10^6$ cells into 6-cm diameter tissue culture dishes. To generate single prominin-1^+ cell-derived clones, prominin-1^+ cells from lung explants of C57Bl/6-EGFP mouse were sorted with magnetic beads as described above and 1 to 5 sorted cells were plated on non-transgenic lung-derived feeder layer in the CEM. For type II lung alveolar epithelial differentiation, cells were cultured in the modified Small Airway Growth Medium (SAGM; Cambrex) as described previously [1]. Macrophage differentiation was induced with 10 ng/mL macrophage-colony stimulating factor (M-CSF, PeproTech) in the CEM. 10 ng/mL TGF-β (PeproTech) was added to the CEM to stimulate myofibroblast differentiation. All cells were incubated at 37°C in a humidified atmosphere containing 5% CO_2. The culture medium was changed two to three times a week.

Reverse Transcription and Real-Time Polymerase Chain Reaction

RNA isolation and cDNA synthesis were performed as described [1]. cDNA was amplified using the Power SYBR Green PCR Master Mix (Applied Biosystems) and oligonucleotides complementary to transcripts of the analyzed genes using the 7500 Fast Real-Time PCR System (Applied Biosystems). The following oligonucleotides were used in this study: αSMA (*Acta2)*: 5'-cgctgtcaggaaccctgaga-3', 5'-cgaagccggccttacaga-3'; collagen I (*Col1a1)*: 5'-gatgacgtgcaatgcaatgaa-3', 5'-ccctcgactcctacatcttctga-3'; fibronectin (*Fn1)*: 5'-taccaaggtcaatccacacccc-3', 5'-cagatggcaaaagaaagcagagg-3'; gapdh (*Gapdh)*: 5'-ctgcaccaccaactgcttagc-3', 5'-ggcatggactgtggtcatgag-3'; SP-C (*Sftpc)*: 5'-tatgactaccagcggctcct-3', 5'-gtttctaccgaccctgtgga-3'. Transcript levels of *Gapdh* were used as endogenous reference, and relative gene expression was analyzed using the $2^{-\Delta\Delta Ct}$ methods.

Histology, immunocytochemistry and phagocytosis assay

Formalin-fixed, paraffin-embedded lung sections were stained with hematoxylin and eosin for histological analysis and with Masson's trichrome staining for detection of collagen fibers. The degree of fibrosis, analyzed as collagen I depositions, was calculated as percentage of the fibrotic area in relation to the total lung area. Calculations represent the average of 5 independent sections for each studied lung tissue.

Immunofluorescence analysis was performed on frozen tissue sections and cells cultured on gelatin-coated cover slips as described previously [1]. For prominin-1 detection, frozen sections and cultured cells were stained with the appropriate primary antibody

prior to fixation with 4% paraformaldehyde. The following primary, secondary antibodies and dilutions were used in this study: rat-anti-prominin-1 1:200, anti–prominin-1-PE 1:200 (eBioscience), anti-F4/80-PE 1:400, anti-Cxcr4-FITC 1:400 (both from BD Bioscience), rabbit anti–surfactant protein-C 1:400 (SP-C, Abcam), mouse anti-β-tubulin IV 1:400 (Sigma), rabbit anti-collagen I 1:400 (Sigma), rabbit anti-fibronectin 1:400 (Milipore), mouse anti-smooth muscle actin 1:1000 (Sigma), donkey anti-rat AlexaFluor488 1:400, chicken anti-rabbit Alexa Fluor 488 1:400, chicken anti-rabbit Alexa Fluor 546 1:600, and goat anti-mouse Alexa Fluor 546 1:600 (all from Molecular Probes). Phagocytosis activity assay was performed using the Texas Red-conjugated *E. coli* BioParticles (Invitrogen) according to manufacture's recommendations.

Western-blot

Prominin-1$^+$ cells were challenged with TGF-β (PeproTech) for 1, 6 and 24 hours. Control cells were cultured in the absence of TGF-β. Cell lysates were blotted and incubated with rabbit anti-Phospho-Smad2 (1:500) and rabbit anti-Smad2/3 (1:1000; both Cell Signaling Technology), and β-actin (1:20000, Sigma).

Flow cytometry

Cells were filtered through 70-μm nylon mesh filter, stained for 30 minutes on ice with the appropriate antibodies, and analyzed on a CyAN ADP (Dako-Cytomation) using FlowJo 8.7.3 software (TreeStar). The following mouse specific antibodies and dilutions were used: anti–prominin-1-PE 1:200, biotin anti–c-kit 1:200, biotin anti-CD45 1:400 (all eBioscience), anti-F4/80-PE 1:400 and streptavidin-APC 1:600 (both BD Bioscience).

References

1. Germano D, Blyszczuk P, Valaperti A, Kania G, Dirnhofer S, Landmesser U, Luscher TF, Hunziker L, Zulewski H, Eriksson U: Prominin-1/CD133+ lung epithelial progenitors protect from bleomycin-induced pulmonary fibrosis. *Am J Respir Crit Care Med* 2009, 179(10):939-949.

Additional Figure legends

Figure S1. Prominin-1$^+$ progenitors protect from induction, but do not affect progression of bleomycin-induced experimental pulmonary fibrosis

Administration of prominin-1$^+$ cells 2 hours after bleomycin treatment protected the mice from fibrosis and collagen I deposition in contrast to later cell administration (24h, 3d, 14d). The degree of fibrosis, analysed as collagen I depositions, was calculated as percentage of the fibrotic area (collagen I-positive area) in relation to the total lung area. Lung tissue sections were stained with Masson's trichrome and analyzed at day 21 after injections of prominin-1$^+$ cells 2 or 24 hours, and 3 or 14 days after bleomycin (BLM) instillation. Control healthy mice (PBS) or bleomycin-challenged mice (BLM) were injected only with saline (PBS). Calculations represent the average of 5 independent sections for each studied lung tissue. Differences were considered as statistically significant for $p<0.05$ (*), $p<0.01$ (**), $p<0.001$ (***).

Figure S2. The prominin-1 expression correlates with the disease state of the bleomycin-treated mice.

A, Immunofluorescent of healthy lung section (d0) showed the distinct cellular distribution of prominin-1- and αSMA-expressing cells. **B**, Upon bleomycin (BLM) treatment the mice develop pulmonary inflammation (d7) characterized by the massive cell infiltration within the lung tissue. At that stage prominin-1-expressing, but αSMA-negative cells represent an abundant fraction. **C-D**, Thorough the chronic stage prominin-1 expression was down-regulated whereas αSMA$^+$ fibroblasts become the

major cellular component of the lung tissue. **A-D,** DAPI visualized cell nuclei. Bars = 20μm.

Figure S3. Bone marrow-derived prominin-1$^+$ cells accumulated within the inflamed lung.

C57Bl/6 mice were lethally irradiated and reconstituted with bone marrow of syngeneic C57Bl/6-EGFP animals. 6 weeks after bone marrow reconstitution, chimeric mice received bleomycin to induce pulmonary fibrosis. In the chimeric mice 7 days after bleomycin (BLM) instillation, EGFP-positive cells accumulated in the inflamed lung tissue, and around 30-40% expressed prominin-1 (**A**). At day 21 after the bleomycin treatment the microphotograph of the lung from the chimeric mice showed no co-localization of EGFP and β-tubulin IV (**B**). DAPI visualized cell nuclei. Bars = 20μm.

Figure S4. Chimeric mice develop BLM-induced pulmonary fibrosis.

Hematoxylin and eosin (H&E)–stained histopathological sections and Masson's Trichrome staining for collagen I deposition from lungs of healthy (d0) C57Bl/6 mice (**A**) and of animals at day 7 (**B**) and 21 (**C**) after bleomycin (BLM) exposure. Original magnifications x100 (for H&E and Masson's trichrome).

Figure S5. TGF-β mediates fibroblast differentiation of lung inflammatory prominin-1$^+$ cells *in vitro*.

Western blotting analysis of Smad2 phosphorylation (P-Smad2) in lung inflammatory prominin-1$^+$ cells cultivated *in vitro* in the presence of TGF-β for 0, 1, 6 and 24 hours.

Samples from two independent experiments are shown for each time point. In total n= 4.

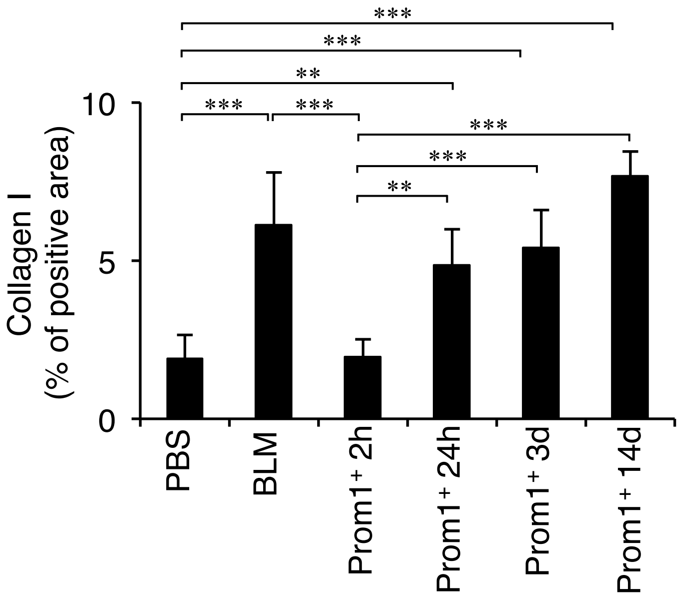

***
***
**
***
***
***
***
**
10
5
0
Collagen I
(% of positive area)
PBS
BLM
Prom1+ 2h
Prom1+ 24h
Prom1+ 3d
Prom1+ 14d

Figure S1

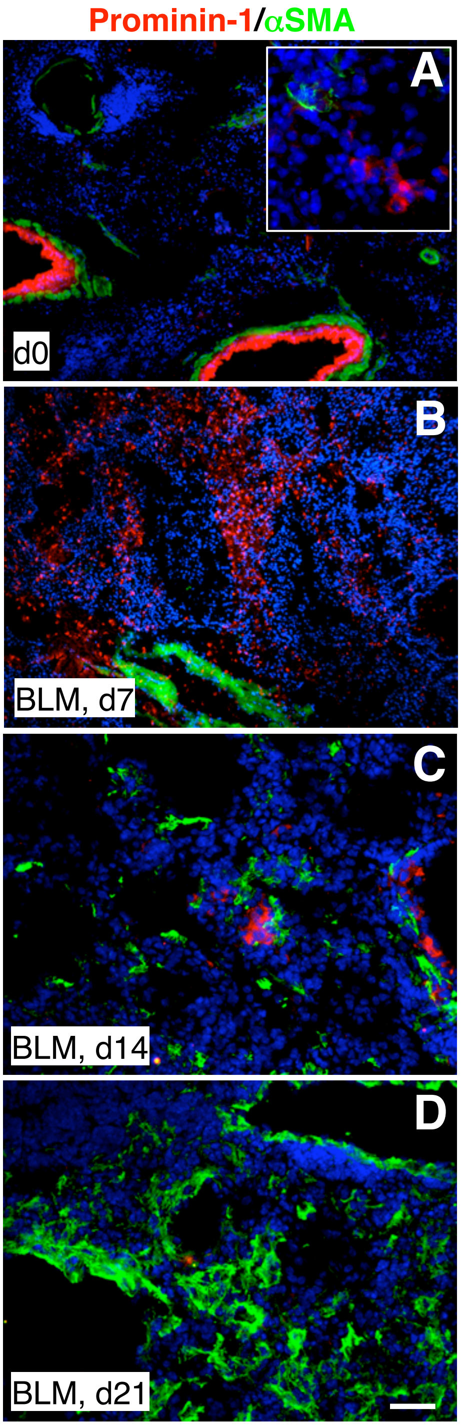

Figure S2

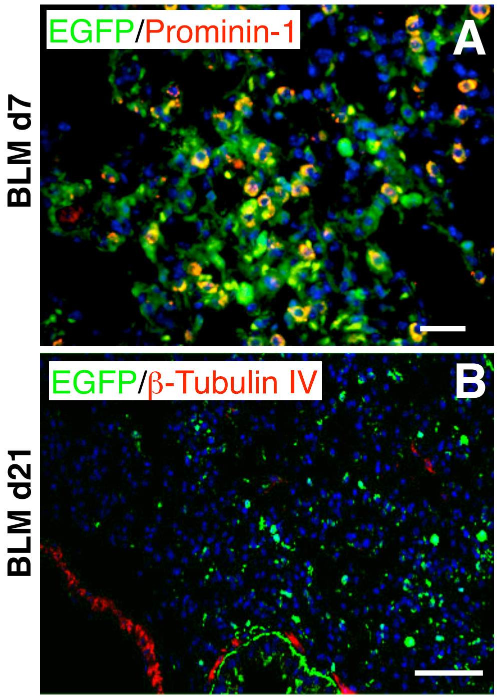

Figure S3

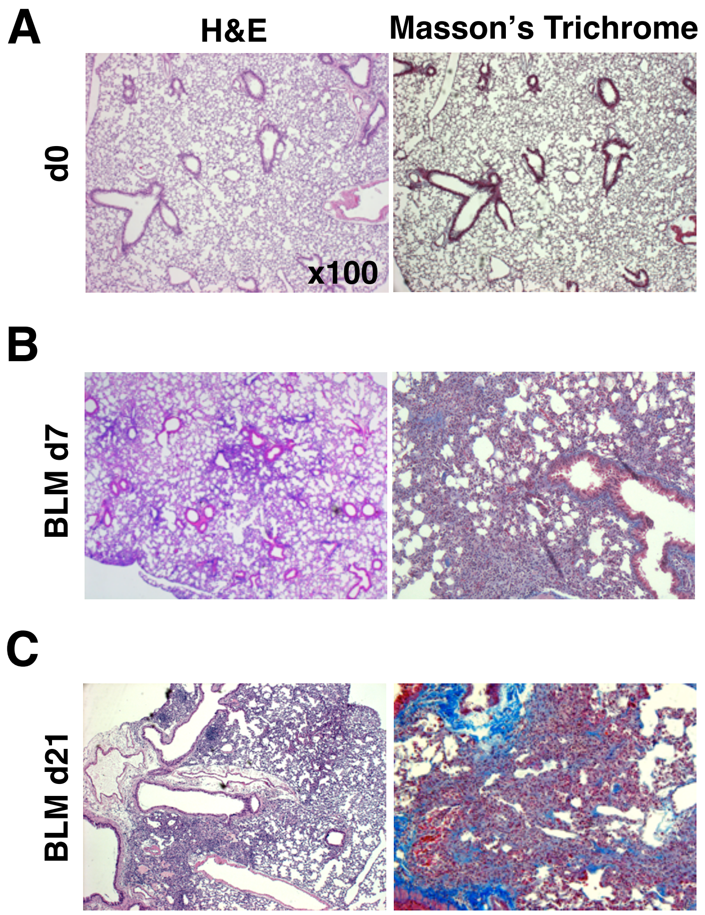

Figure S4

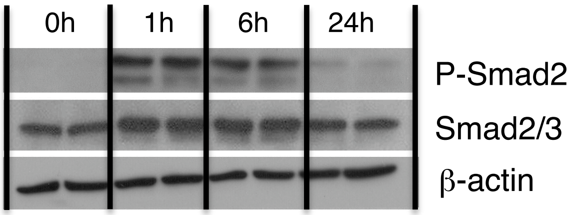

Figure S5
